# Inferior vena cava filters in the intensive care unit population: single center experience in the united arab emirates

**DOI:** 10.1186/2197-425X-3-S1-A970

**Published:** 2015-10-01

**Authors:** AS Beshyah, SA AlBhaisi, K Krishnanreddy, A Khwaja

**Affiliations:** Sheikh Khalifa Specialty Hospital, Internal Medicine, Abu Dhabi, United Arab Emirates; Sheikh Khalifa Specialty Hospital, Intensive Care Unit, Abu Dhabi, United Arab Emirates; Sheikh Khalifa Specialty Hospital, Interventional Radiology, Abu Dhabi, United Arab Emirates

## Introduction

Pharmacological prophylaxis against venous thromboembolism using low molecular weight heparin (LMWH) has become a standard measure in the intensive care unit (ICU) [1–3]. Risk factors in these patients include critical illness, mechanical ventilation, sedative medications and central venous catheter insertion [2]. In cases where pharmacological prophylaxis is not feasible, inferior vena cava filters (IVCF) have been recommended [1].

## Objectives

Evaluation of the indications, course and outcome for filter placement in the critically ill population.

## Methods

We retrospectively reviewed charts of 95 patients who had an IVC filter placed between January 2011 and December 2014 at our institution. We studied the indications for IVCF placement, hospital course, insertion/retrieval dates, contraindications to anticoagulation and the complications associated with the filter. These patients were matched to their appropriate IVC filter guideline indications [4], which were analysed.

## Results

53 of the 95 patients were admitted to ICU with a median age of 47 (20-86) years. Of the total ICU population with placement, 37 (70%) of the placements were therapeutic and 16 (30%) were prophylactic. In trauma patients (N-20), 70% of the IVC filters were placed as a prophylactic measure whereas in non-traumatic cases 94% of IVC filters were placed for therapeutic indications. Venous access was mostly via the right internal jugular vein (91%) and majority were placed infra-renal (96%). 16 IVC's were retrieved after a median of 67 (21-185) days; representing 30% of the total and 41% of the surviving patients. No immediate procedural complications occurred during placement or retrieval; 3 developed DVT and 1 patient developed PE after insertion. Of the total population involved, 14 patients (26%) died (all being in the non-trauma subgroup). 8 patients were lost to follow up.

## Conclusions

Our review shows that the IVCF practices at our institution are consistent with the accepted recommendations. The vast majority of the patients had a contraindication to anticoagulation therapy. The rate of immediate and delayed complications are low, however further follow up is required to assess the incidence of late complications. All patients who died after placement of an IVC were non-trauma patients with serious co-morbidities, which should allow us to be more liberal in their use in trauma cases.

**Table 1:** Table 1

Mean Admission Days (Total population)	67.4
Shortest Admission (Total population)	1
Longest Admission (Total population)	243
Mean Admission Days (Trauma)	19.1
Shortest Admission (Trauma)	2
Longest Admission (Trauma)	76
Mean Admission Days (Non-trauma)	28.7
Shortest Admission (Non-trauma)	1
Longest Admission (Non-trauma)	243

*[ICU Admission Data]*

**Figure 1:**
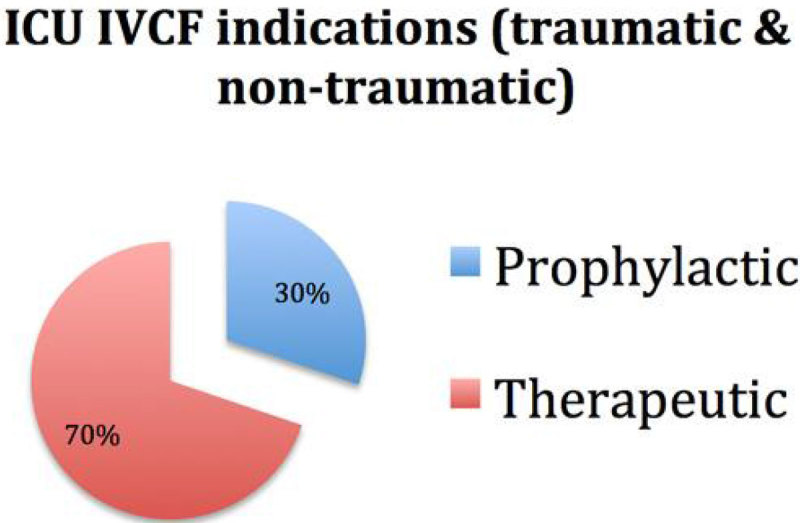
**ICU indications trauma and non-trauma**.

**Figure 2:**
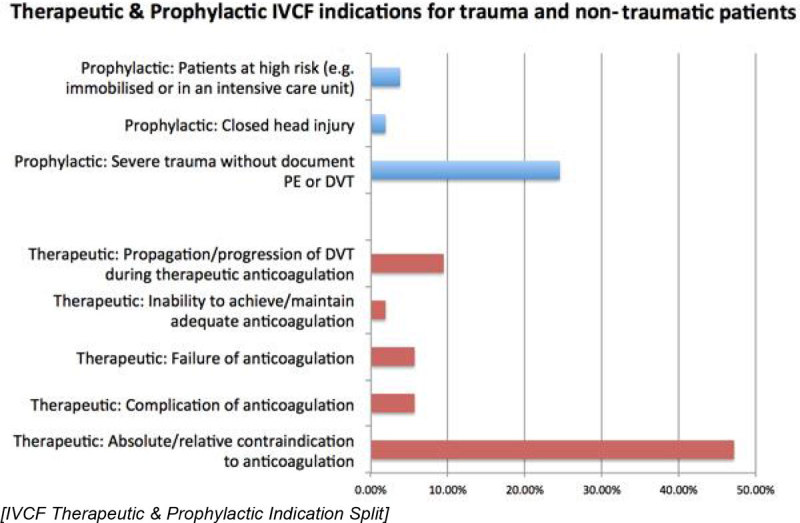
**IVCF Therapeutic & Prophylactic Indication Split**.

**Figure 3:**
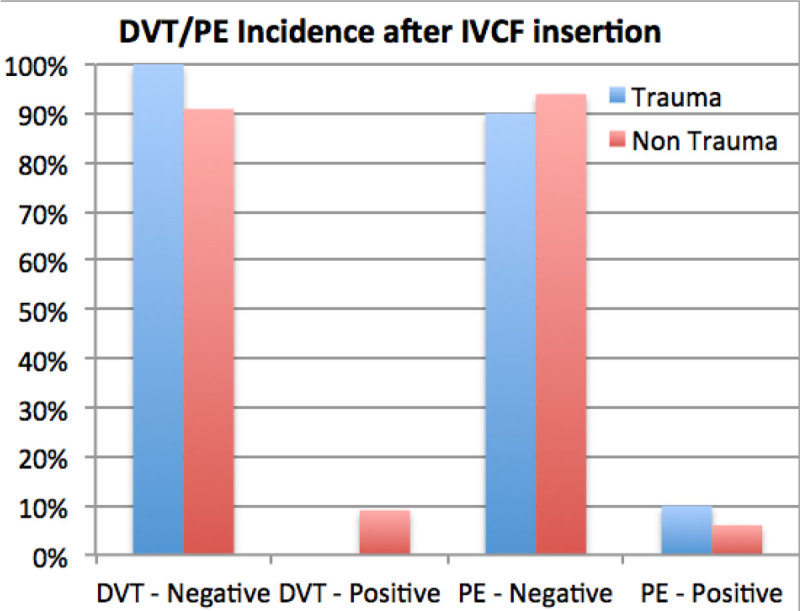
**DVT & PE Incidence after IVCF insertion**.
